# Examination of the relationship between perceived freedom levels in leisure time and multidimensional leadership orientations of individuals engaged in nature sports as a recreational activity

**DOI:** 10.3389/fpsyg.2025.1525582

**Published:** 2025-07-14

**Authors:** Kenan Koç, Mustafa Soner Yüce, Hakkı Ulucan, Mustafa Zahit Serarslan, Oğulcan Usuflu, Sevim Kir, Erdal Kirik

**Affiliations:** ^1^Faculty of Sports Sciences, Erciyes University, Kayseri, Türkiye; ^2^Department of Physical Education and Sports, Institute of Health Sciences, Erciyes University, Kayseri, Türkiye; ^3^Faculty of Sports Sciences, Istanbul Sabahattin Zaim University, Istanbul, Türkiye

**Keywords:** recreation, nature sports, perceived freedom, leadership, leisure time

## Abstract

**Introduction:**

The purpose of this study is to examine the relationship between perceived freedom in leisure and multifaceted leadership orientations among individuals engaged in recreational nature sports residing in various provinces of the Central Anatolia region.

**Methods:**

In this study, a quantitative research method was used. The sample consisted of 633 randomly selected volunteers from Kayseri, Kýrşehir, and Nevşehir. Data collection instruments included the “Multifaceted Leadership Orientations Scale”, “Perceived Freedom in Leisure Scale”, and demographic information form. Participants' personal information, inventory scores, and factor scores were presented using frequency (f) and percentage (%) values. For comparing scale scores, independent *t*-test statistics were used for gender and marital status variables, while one-way analysis of variance (LSD) was employed for age, educational status, sports discipline, duration of engagement in nature sports, and welfare level comparisons. Pearson correlation analysis (r) was applied to determine the relationship between the scores obtained from the scales.

**Results:**

The research findings indicate that individuals participating in recreational nature sports activities exhibit high levels of perceived freedom in leisure and average levels of leadership orientation. On the other hand, analysis of various variables revealed significant differences between perceived freedom in leisure time and marital status, sport discipline, and years of participation, as well as between the some sub-dimensions of multifaceted leadership orientations and gender, marital status, education level, age, sport discipline, years of participation, and welfare level.

**Discussion:**

The correlation analysis revealed a strong positive relationship between perceived freedom in leisure levels and multifaceted leadership orientations. This positive correlation is thought to stem from individuals' desire to prioritize their own wishes, emotions, and ideas to feel free during their leisure time.

## Introduction

Nature sports are defined as any type of sport performed in nature (Ardahan and Lapa, 2010). Nature sports are expressed as activities of struggling against the existing potential difficulties and risks of nature and sustaining life without any motor or animal power support, using only the knowledge, skills, and conditioning possessed by humans. Sporting activities in nature have been classified under various names such as “nature sports,” “outdoor recreation activities,” “adventure sports,” “adventure recreation,” and “extreme sports,” depending on the level of danger they involve, risk factors, and auxiliary elements used (Koçak and Balci, 2010). Moreover, activities in a natural environment have positive roles on the participants' experiences of experiential learning, self-awareness and socialization (Yıldız, 2022).

Today, participation in recreational activities, the desire to connect with nature, and the utilization of leisure time have become a necessity for modern humans. The modern individual, fulfilling their obligations and oppressed under the pressure of daily compulsory actions, obtains renewal within their leisure time through recreational activities in which they participate according to their interests (Bilgen and Yüksel, 2021. Recreation and leisure are fundamentally for relaxation, entertainment, and personal development; as such, activities have emerged that address individual needs, particularly including stress management, freedom, self-esteem, and identity development (Atchley, 1970).

Individuals who experience a high level of perceived freedom in their leisure time perceive themselves as competent and in control of what happens before, during, and after leisure participation. Perceived freedom has been reported as the primary defining criterion of leisure (Ellis, and Ve Witt, 1994). Perceived freedom is a matter of degree. Most of the time, there is some kind of constraint on people's leisure time. Despite these constraints, they experience a sense of freedom in their leisure choices (Mannell and Kleiber, 1997).

Perceived freedom in leisure reflects individuals' self-assessments of their ability to participate in leisure activities and is therefore affected by events occurring in their lives (Yerlisu et al., 2012) According to Zowislo (2010), “What seems striking and meaningful is that everything about human beings actually originates from leisure time.” In other words, people can set recreation and leisure as a goal for themselves and also experience the outcomes that result from achieving this goal. According to Sartre (2011), since human essence is not predetermined, humans will find their own essence and reconstruct themselves through their experiences, actions, and choices, which is only possible as long as humans are free in their actions and choices. In recreational activities, individuals may want to prioritize their own values, ideas, and orientations in order to feel free. At this point, the concept of leadership gains importance within the scope of group recreational activities in which the individual participates.

Leaders are individuals who pursue group goals through personal motivation rather than through coercion (Bolden, 2004). As the concept of recreation has evolved, so too has the idea of recreational leadership (Kirtepe and Ugurlu, 2022), which has become increasingly important today, particularly in supporting individuals facing weakened mental resilience, intense work demands, and experiences of personal control loss and ego burnout (Irhan, 2023). Freedom in leisure time is a fundamental aspect that allows individuals to choose activities voluntarily, fostering a sense of autonomy and personal fulfillment. This perceived freedom enhances motivation and engagement, which are crucial for effective leadership and group dynamics. Recreational leadership refers to the capacity to organize and direct group activities while motivating members within the group (Russell, 2001). A recreation leader is expected to foster a sense of joy in both the group and its tasks, ensure that members enjoy their participation, provide guidance, act as a role model, and influence members by engaging them in various activities (Karaku̧çük et al., 2017). In this way, leadership in leisure activities not only guarantees that recreational pursuits are carried out correctly and healthily but also supports team members in discovering their own essence (Kozak et al., 2017).

This study aims to investigate the relationship between individuals' perceived freedom in leisure time and their multifaceted leadership orientations, focusing specifically on those involved in nature sports for recreational purposes.

## Materials and methods

### Study group

This research employs a correlational survey model. This survey model can be defined as “research models aiming to determine the existence and/or degree of covariance between two or more variables” (Karasar, 2015).

The research carries descriptive characteristics as it aims to establish the relationship between perceived freedom levels in leisure time and multifaceted leadership orientations among individuals engaging in recreational outdoor sports residing in different provinces of the Central Anatolia region. The Central Anatolia Region is located in the heart of Turkey and is characterized by vast and diverse natural areas. This region offers significant opportunities for nature sports, with activities such as cycling, mountaineering, paragliding, orienteering, camping, trekking, and canoeing being widely practiced. The rugged terrain, open spaces, and lakes of Central Anatolia provide a suitable environment for nature sports enthusiasts, contributing to the development of these activities in the region.

### Inclusion and exclusion in the study

The study included individuals aged 18 and above who regularly participate in nature sports for recreational purposes. Participants were required to engage consistently in activities such as cycling, mountaineering, paragliding, orienteering, camping, trekking, and canoeing. Those excluded from the study were individuals under the age of 18, those who do not engage in nature sports recreationally, and individuals who did not consent to participate voluntarily. This criteria ensured that the sample consisted of relevant participants whose experiences could accurately reflect the study's objectives.

### Data collection tools

During the administration of the surveys, the researchers aimed to provide an adequate evaluation process for each participant within a sufficient time frame, ensuring that no rush occurred and necessary explanations were given. Moreover, appropriate conditions were created to allow participants to complete the forms in a comfortable environment. On average, it took participants ~10 min to complete the scales. The data collection instruments used in this study included the “Multifaceted Leadership Orientations Scale” developed by Dursun et al. (2019), the “Perceived Freedom in Leisure Scale” developed by Yerlisu Lapa and Tercan Kaas (2019), and a socio-demographic information form.

### Formation of volunteer groups

The research was conducted through a study group. The study group was selected using a convenience sampling method. The study group comprised individuals residing in different provinces of the Central Anatolia region. A total of 633 individuals participated in the study. The study was conducted in three provinces within the Central Anatolia Region of Turkey: Kayseri, Kirşehir, and Nevşehir. Participants were reached through recreational sports clubs, local outdoor sports organizations, and public activity areas commonly used for nature sports. Convenience sampling was employed, and voluntary participation was encouraged through direct contact and informational briefings. The sample size was determined based on the accessible population in the selected provinces, using standard formulas for quantitative research to ensure statistical significance and representation. All participants took part in the study voluntarily and were informed about the purpose and confidentiality of the research prior to their participation. Data collection was carried out between January and March 2024.

### Demographic information form

In developing the demographic information form, research studies containing multifaceted leadership orientations and perceived freedom in leisure scales, along with socio-demographic information forms in the literature, were examined, and a pool of characteristics to be identified in athletes was created. Subsequently, with assistance from statistical experts, the socio-demographic information form was developed. This form contains seven questions designed to obtain information about gender, marital status, age, educational status, sports discipline of interest, duration of engagement in outdoor sports, and welfare level.

### Multifaceted Leadership Orientations Scale (MLOS)

The Multifaceted Leadership Orientations Scale, developed by Dursun et al. (2019), is designed to measure individuals' leadership tendencies across four key dimensions: Political Leadership, Human-Oriented Leadership, Charismatic Leadership, and Structural Leadership. The scale consists of 19 items distributed as follows: Political Leadership (5 items), Human-Oriented Leadership (5 items), Charismatic Leadership (5 items), and Structural Leadership (4 items). Each item is rated on a 5-point Likert scale ranging from 1 (Strongly Disagree) to 5 (Strongly Agree), with higher scores indicating a stronger inclination toward the respective leadership style. The scale contains no reverse-scored items. It aims to reflect the multifaceted nature of leadership by assessing how individuals display different leadership styles in varying contexts. The internal consistency coefficients (Cronbach's alpha) reported by the original authors were 0.80 for Political Leadership, 0.73 for Human-Oriented Leadership, 0.74 for Charismatic Leadership, and 0.72 for Structural Leadership, with an overall reliability of 0.85. In the current study, the overall reliability was found to be 0.90, with sub-dimension coefficients of 0.73 for Political Leadership, 0.77 for Human-Oriented Leadership, 0.76 for Charismatic Leadership, and 0.78 for Structural Leadership, demonstrating high internal consistency.

### Perceived Freedom in Leisure Scale

The “Perceived Freedom in Leisure Scale” developed by Yerlisu Lapa and Tercan Kaas (2019) was adapted as a 5-point Likert format: Strongly disagree (1), Disagree (2), Undecided (3), Agree (4), Strongly agree (5). The scale provides scores based on the mean score, with all items being positively worded. The mean score is calculated by summing item scores and dividing by the number of items, with higher mean scores indicating greater perceived freedom in leisure time. The scale is unidimensional, consisting of 25 items, with a Cronbach's Alpha coefficient of 0.93.

### Data analysis

Personal information, inventory scores, and factor scores were analyzed using frequency (f) and percentage (%) values. The distribution of scores was examined using Skewness-Kurtosis values. The data distribution was determined to be within the ±1 range, which Büyüköztürk (2007) interprets as appropriate for normality. Consequently, parametric test statistics were employed for data comparison. Independent T-tests were utilized for binary comparisons, while one-way analysis of variance was applied for comparing three or more variables. For sub-dimensions showing significant differences in one-way analysis of variance, LSD test statistics were employed for paired comparisons in cases of homogeneous distribution and unequal group numbers. Pearson Product-Moment Correlation analysis (r) was conducted to determine relationships between scale scores. Table 1 shows the socio-demographic characteristics of the participants.

**Table 1:** Socio-demographic characteristics of participants.

**Socio-demographic characteristics**	**Variables**	***N***	**%**
Gender	Male	343	54.2
	Female	290	45.8
Marital status	Married	283	44.7
	Single	350	55.3
Age	18–28	113	17.9
	29–39	245	38.7
	40–50	144	22.7
	51 and above	131	20,7
Educational status	High School	121	19.1
	Associate/Bachelor's Degree	427	67.5
	Graduate Degree	85	13.4
Branches	Cycling	56	8.8
	Mountaineering	103	16.3
	Paragliding	108	17.1
	Orienteering	100	15.8
	Camping	124	19.6
	Trekking	91	14.4
	Canoeing	51	8.1
Individuals' years of participation in recreational activities	0–2	93	14.7
	3–5	193	30.5
	6–8	238	37.6
	9 and above	109	17.2
Welfare level	Poor	77	12.2
	Moderate	169	26.7
	Good	279	44.1
	Very good	108	17.1

## Findings

Table 2 shows that for individuals engaged in nature sports as a recreational activity, the mean subdimension score of the perceived freedom in leisure scale is 101.79 ± 5.79. For the multifaceted leadership orientations scale, the mean scores are as follows: political leadership subdimension 15.11 ± 3.95, human resource leadership subdimension 15.60 ± 3.98, charismatic leadership subdimension 15.63 ± 4.23, structural leadership subdimension 12.94 ± 3.18, and multifaceted leadership subdimension 77.24 ± 4.81.

**Table 2:** Descriptive statistics of scores obtained by individuals from perceived freedom in leisure and multifaceted leadership orientations scales.

**Scale**	***N***	**Minimum**	**Maximum**	**M ±Sd**	**Skewness**	**Kurtosis**
Perceived freedom in leisure	633	84.00	114.00	101.79 ± 5.79	−0.318	−0.279
Political leadership	633	5.00	25.00	15.11 ± 3.95	0.305	−0.307
Human resource leadership	633	6.00	25.00	15.60 ± 3.98	0.126	−0.634
Charismatic leadership	633	5.00	25.00	15.63 ± 4.23	0.133	−0.508
Structural leadership	633	5.00	20.00	12.94 ± 3.18	0.050	−0.389
Multifaceted leadership	633	62.00	88.00	77.24 ± 4.81	0.347	0.215

M, Mean; Sd, Standard Deviation.

Table 3 presents a comparison of perceived freedom in leisure and multifaceted leadership orientations scale scores according to individuals' gender. No significant difference was observed between male (*N* = 343) and female (*N* = 290) participants in terms of perceived freedom in leisure levels (t = −0.569, *p* = 0.573). However, in political leadership orientations, males (X ± SD: 15.42 ± 3.90) scored significantly higher than females (X ± SD: 14.78 ± 3.97) (t = 0.970, *p* = 0.043), indicating that males have stronger political leadership orientations than females. A similar trend is observed in human resource leadership, with a significant difference (t = 0.890, *p* = 0.007) between males (X ± SD: 15.99 ± 3.93) and females (X ± SD: 15.14 ± 3.94), suggesting that males have a more dominant attitude in human resource leadership. In charismatic leadership, males (X ± SD: 16.11 ± 4.07) again scored significantly higher than females (X ± SD: 15.06 ± 4.33) (t = 0.192, *p* = 0.002). No significant difference was found in structural leadership orientations between genders (t = 0.700, *p* = 0.353). Overall, the comparison between genders reveals that males score higher in political, human resource, and charismatic leadership orientations compared to females, while they are at similar levels in perceived freedom in leisure and structural leadership.

**Table 3:** Comparison of perceived freedom in leisure and multifaceted leadership orientations scale scores according to individuals' gender.

**Subdimensions**	**Gender**	***N***	**M ±Sd**	**t**	***p***
Perceived freedom in leisure	Male	343	101.66 ± 5.42	−0.569	0.573
	Female	290	101.92 ± 6.15		
Political leadership	Male	343	15.42 ± 3.90	0.970	***0.043***
	Female	290	14.78 ± 3.97		
Human resource leadership	Male	343	15.99 ± 3.93	0.890	***0.007***
	Female	290	15.14 ± 3.94		
Charismatic leadership	Male	343	16.11 ± 4.07	0.192	***0.002***
	Female	290	15.06 ± 4.33		
Structural leadership	Male	343	12.83 ± 3.14	0.700	0.353
	Female	290	13.07 ± 3.21		

M, Mean; Sd, Standard Deviation; t, *t*-value; p, *p*-value. *p* < 0.05. Bold numbers are used to emphasize significant differences.

Table 4 presents a comparison of the effects of marital status on perceived freedom in leisure and multifaceted leadership orientations. The results indicate that married individuals' mean score for perceived freedom in leisure (X = 100.92) is significantly lower than that of single individuals (X = 102.47) (t = −3.391; *p* = 0.001). Furthermore, in the assessments conducted among leadership orientations, married individuals obtained higher scores compared to single individuals in the areas of political leadership (t = 0.034; *p* = 0.005), human resource leadership (t = 0.080; *p* = 0.001), and charismatic leadership (t = 2.272; *p* = 0.006), while no significant difference was observed in the area of structural leadership (t = 0.139; *p* = 0.587). These results suggest that married individuals may be more successful or effective in certain leadership orientations, while also revealing that the perception of leisure time may be negatively affected by marital status.

**Table 4:** Comparison of perceived freedom in leisure and multifaceted leadership orientations scale scores according to individuals' marital status.

**Subdimensions**	**Marital status**	***N***	**M ±Sd**	**t**	***p***
Perceived freedom in leisure	Married	283	100.92 ± 5.11	−3.391	***0.001***
	Single	350	102.47 ± 6.16		
Political leadership	Married	283	15.62 ± 3.67	2.837	***0.005***
	Single	350	14.72 ± 4.11		
Human resource leadership	Married	283	16.20 ± 3.73	3.460	***0.001***
	Single	350	15.12 ± 4.07		
Charismatic leadership	Married	283	16.14 ± 4.02	2.272	***0.006***
	Single	350	15.22 ± 4.34		
Structural leadership	Married	283	12.96 ± 3.06	0.139	0.587
	Single	350	12.92 ± 3.27		

M, Mean; Sd, Standard Deviation; t, *t*-value; p, *p*-value. *p* < 0.05. Bold numbers are used to emphasize significant differences.

Table 5 compares perceived freedom in leisure and multifaceted leadership orientation scale scores across age groups, examining statistical differences between age categories. The scores for perceived freedom in leisure averaged 102.44 (SD = 5.66) in the 18–28 age group, while this value was observed as 100.83 (SD = 6.16) in the 51 and above age group. In the political leadership subdimension, the highest score was recorded in the 40–50 age group (15.91; SD = 3.96); however, no statistically significant difference emerged (*p* = 0.053). The human resources leadership analysis revealed that the 18–28 age group demonstrated the lowest mean value of 14.61 (SD = 3.70), while the 29–39 age group showed a significantly higher mean (15.93; SD = 4.04; *p* = 0.007). Furthermore, there was a significant difference with the 40–50 age group (16.10; SD = 3.96). Regarding charismatic leadership and structural leadership dimensions, no significant differences were found among age groups. Overall, the results indicate that there are variations in leadership perception across age groups, although these differences become more pronounced in specific leadership types within certain age brackets.

**Table 5:** Comparison of perceived freedom in leisure and multifaceted leadership orientation scale scores according to individuals' ages.

**Subdimensions**	**Age**	***N***	**M ±Sd**	**f**	***p***	**LSD**
Perceived freedom in leisure	18–28^a^	113	102.44 ± 5.66	2.091	0.100	***-***
	29–39^b^	245	102.13 ± 5.58			
	40–50^c^	144	101.52 ± 5.70			
	51 and above^d^	131	100.83 ± 6.16			
Political leadership	18–28^a^	113	14.96 ± 3.86	2.581	0.053	***-***
	29–39^b^	245	14.80 ± 3.99			
	40–50^c^	144	15.91 ± 3.96			
	51 and above^d^	131	14.98 ± 3.83			
Human resource leadership	18–28^a^	113	14.61 ± 3.70	4.063	***0.007***	***a < b a < c***
	29–39^b^	245	15.93 ± 4.04			
	40–50^c^	144	16,10 ± 3,96			
	51 and above^d^	131	15,28 ± 3,85			
Charismatic leadership	18–28^a^	113	14,84 ± 3,53	2.332	0.073	***-***
	29–39^b^	245	15,56 ± 4,05			
	40–50^c^	144	16,21 ± 5,03			
	51 and above^d^	131	15.81 ± 4.04			
Structural leadership	18–28^a^	113	12.99 ± 2.90	1.013	0.387	***-***
	29–39^b^	245	12.95 ± 3.16			
	40–50^c^	144	13.22 ± 3.71			
	51 and above^d^	131	12.56 ± 2.74			

M, Mean; Sd, Standard Deviation; f, F-value; p, *p*-value; LSD, Least Significant Difference. *p* < 0.05. Bold numbers are used to emphasize significant differences. Letters were used to indicate the differences between the variables on the left side of the tables.

Table 6 presents a comparative analysis of perceived freedom in leisure and multifaceted leadership orientation scale scores according to individuals' educational status. Overall, no significant difference is observed in perceived freedom in leisure levels, with no statistically significant variation (*p* = 0.131) among high school (M = 102.69), associate/bachelor's degree (M = 101.63), and graduate degree (M = 101.22) groups. However, in terms of leadership orientations, the high school group's mean score in political leadership (M = 14.05) falls below both associate/bachelor's degree (M = 15.35) and graduate degree (M = 15.46) groups, with this difference being statistically significant (*p* = 0.005). Regarding human resources leadership, the high school group's score (M = 14.98) was found to be lower than both associate/bachelor's degree (M = 15.58) and graduate degree (M = 16.58) groups, which is also statistically significant (*p* = 0.017). In charismatic leadership, the high school group's score (M = 14.34) is significantly lower compared to other groups (*p* = 0.001). Similarly, in the structural leadership dimension, the high school group's mean (M = 12.01) remains below both associate/bachelor's degree (M = 12.89) and graduate degree (M = 13.08) groups, showing a significant difference (*p* = 0.002). These findings clearly demonstrate the effects of educational level on leadership orientations, indicating that high school graduates exhibit lower levels of leadership characteristics.

**Table 6:** Comparison of perceived freedom in leisure and multifaceted leadership orientation scale scores according to individuals' educational status.

**Subdimensions**	**Educational status**	***N***	**M ±Sd**	**f**	***p***	**LSD**
Perceived freedom in leisure	High School^a^	121	102.69 ± 5.71	2.039	0.131	***-***
	Associate/Bachelor's Degree^b^	427	101.63 ± 5.82			
	Graduate Degree^c^	85	101.22 ± 5.45			
Political leadership	High School^a^	121	14.05 ± 3.82	5.414	***0.005***	***a < b*** ***a < c***
	Associate/Bachelor's Degree^b^	427	15.35 ± 3.97			
	Graduate Degree^c^	85	15.46 ± 3.77			
Human resource leadership	High School^a^	121	14.98 ± 3.50	4.106	***0.017***	***a < c*** ***b < c***
	Associate/Bachelor's Degree^b^	427	15.58 ± 3.99			
	Graduate Degree^c^	85	16.58 ± 4.25			
Charismatic leadership	High School^a^	121	14.34 ± 4.08	7.718	***0.001***	***a < b*** ***a < c***
	Associate/Bachelor's Degree^b^	427	15.84 ± 4.09			
	Graduate Degree^c^	85	16.41 ± 4.71			
Structural leadership	High School^a^	121	12.01 ± 2.75	6.127	***0.002***	***a < b***
	Associate/Bachelor's Degree^b^	427	12.89 ± 3.20			
	Graduate Degree^c^	85	13.08 ± 3.39			

M, Mean Sd; Standard Deviation; f, F-value; p, *p*-value; LSD, Least Significant Difference. *p* < 0.05. Bold numbers are used to emphasize significant differences. Letters were used to indicate the differences between the variables on the left side of the tables.

Table 7 presents a comparative analysis of perceived freedom in leisure and multifaceted leadership orientation scale scores across various recreational activity branches. When examining the scores for perceived freedom in leisure, the canoeing branch demonstrates the highest mean score (M = 103.65), while the orienteering branch shows the lowest mean (M = 98.99), with the difference being statistically significant (*p* < 0.001). While no significant differences were found among branches in political leadership and human resources leadership dimensions, in the charismatic leadership dimension, the orienteering branch exhibits the highest score (M = 17.04), while the trekking branch shows the lowest score (M = 14.34), with results being statistically significant (*p* < 0.001). In the structural leadership dimension, orienteering (M = 13.96) achieves the highest mean score, while the canoeing branch demonstrates the lowest mean score (M = 12.04). Overall, the analysis reveals that different recreational branches have significant effects on individuals' perception of leisure time and leadershistyles.

**Table 7:** Comparison of perceived freedom in leisure and multifaceted leadership orientation scale scores according to individuals' recreational activity branches.

**Subdimensions**	**Branches**	***N***	**M ±Sd**	**f**	***p***	**LSD**
Perceived freedom in leisure	Cycling^a^	56	103.00 ± 6.09	5.709	*** < 0.001***	***a>d*** ***b>d*** ***b < g*** ***c>d*** ***e>d*** ***f>d*** ***g>d***
	Mountaineering^b^	103	101.72 ± 5.47			
	Paragliding^c^	108	102.07 ± 3.73			
	Orienteering^d^	100	98.99 ± 6.73			
	Camping^e^	124	102.11 ± 6.16			
	Trekking^f^	91	102.31 ± 5.49			
	Canoeing^g^	51	103.65 ± 5.38			
Political leadership	Cycling^a^	56	15.04 ± 4.56	1.874	0.083	***-***
	Mountaineering^b^	103	15.09 ± 3.70			
	Paragliding^c^	108	15.78 ± 3.96			
	Orienteering^d^	100	14.79 ± 4.11			
	Camping^e^	124	15.41 ± 4.46			
	Trekking^f^	91	14.12 ± 2.23			
	Canoeing^g^	51	15.65 ± 4.17			
Human resource leadership	Cycling^a^	56	15.54 ± 4.38	1.350	0.233	***-***
	Mountaineering^b^	103	15.59 ± 4.22			
	Paragliding^c^	108	15.98 ± 4.00			
	Orienteering^d^	100	16.11 ± 3.94			
	Camping^e^	124	15.41 ± 4.12			
	Trekking^f^	91	14.70 ± 3.10			
	Canoeing^g^	51	15.96 ± 3.70			
Charismatic leadership	Cycling^a^	56	15.05 ± 4.58	3.892	***0.001***	***a < d*** ***b < d*** ***c>f*** ***d>a*** ***d>b*** ***d>e*** ***d>f*** ***d>g*** ***e>f***
	Mountaineering^b^	103	15.41 ± 4.71			
	Paragliding^c^	108	16.03 ± 4.07			
	Orienteering^d^	100	17.04 ± 3.60			
	Camping^e^	124	15.74 ± 4.04			
	Trekking^f^	91	14.34 ± 3.40			
	Canoeing^g^	51	15.14 ± 5.16			
Structural leadership	Cycling^a^	56	12.29 ± 4.07	4.715	*** < 0.001***	***a < c*** ***a < d*** ***b < c*** ***b < d*** ***b < e*** ***b < f*** ***c>g*** ***d>e*** ***d>g*** ***f>g***
	Mountaineering^b^	103	12.07 ± 3.11			
	Paragliding^c^	108	13.43 ± 3.30			
	Orienteering^d^	100	13.96 ± 2.74			
	Camping^e^	124	12.90 ± 3.02			
	Trekking^f^	91	13.15 ± 2.65			
	Canoeing^g^	51	12.04 ± 3.27			

M, Mean Sd; Standard Deviation; f, F-value; p, *p*-value; LSD, Least Significant Difference. *p* < 0.05. Bold numbers are used to emphasize significant differences. Letters were used to indicate the differences between the variables on the left side of the tables.

Table 8 examines the differences in perceived freedom in leisure and multifaceted leadership orientations based on individuals' duration of participation in recreational activities. In terms of perceived freedom in leisure, the lowest score (102.25 ± 5.65) was observed among participants with 0–2 years of experience, while those with 9 years and above demonstrated the highest score (103.59 ± 6.48), showing statistical significance (f = 5.982, *p* = 0.001). A similar trend is observed in political leadership, where participants with 0–2 years of experience showed notably lower means (13.76 ± 4.34) compared to other groups (f = 4.986, *p* = 0.002). Regarding human resources leadership, individuals with 0–2 years of participation again demonstrated the lowest scores (14.24 ± 4.07), with a significant difference (f = 5.722, *p* = 0.001). In the charismatic leadership category, no significant differences were found between group scores (p > 0.05). In the domain of structural leadership, those with 0–2 years of participation (12.08 ± 3.21) obtained lower scores compared to other groups, with this difference being statistically significant (f = 6.377, *p* = 0.000). Overall, the findings indicate that as the duration of participation increases, individuals' scores in perceived freedom in leisure and leadership orientations show an upward trend.

**Table 8:** Comparison of perceived freedom in leisure and multifaceted leadership orientation scale scores according to individuals' years of participation in recreational activities.

**Subdimensions**	**Years**	***N***	**M ±Sd**	**f**	***p***	**LSD**
Perceived freedom in leisure	0–2^a^	93	102.25 ± 5.65	5.982	***0.001***	***a < d*** ***b < d*** ***c < d***
	3–5^b^	193	102.36 ± 5.96			
	6–8^c^	238	102.55 ± 5.07			
	9 and above^d^	109	103.59 ± 6.48			
Political leadership	0–2^a^	93	13.76 ± 4.34	4.986	***0.002***	***a < b*** ***a < c*** ***a < d***
	3–5^b^	193	15.03 ± 3.42			
	6–8^c^	238	15.13 ± 4.09			
	9 and above^d^	109	15.39 ± 3.92			
Human resource leadership	0–2^a^	93	14.24 ± 4.07	5.722	***0.001***	***a < b*** ***a < c*** ***b>d***
	3–5^b^	193	16.16 ± 3.55			
	6–8^c^	238	15.85 ± 4.26			
	9 and above^d^	109	15.24 ± 3.57			
Charismatic leadership	0–2^a^	93	14.92 ± 4.38	1.931	0.123	***-***
	3–5^b^	193	15.36 ± 4.40			
	6–8^c^	238	16.03 ± 4.10			
	9 and above^d^	109	15.83 ± 3.95			
Structural leadership	0–2^a^	93	12.08 ± 3.21	6.377	*** < 0.001***	***a < b*** ***a < d*** ***b>c***
	3–5^b^	193	13.59 ± 3.20			
	6–8^c^	238	12.60 ± 3.06			
	9 and above^d^	109	13.27 ± 3.11			

M, Mean; Sd, Standard Deviation; f, F-value; p, *p*-value; LSD, Least Significant Difference. *p* < 0.05. Bold numbers are used to emphasize significant differences. Letters were used to indicate the differences between the variables on the left side of the tables.

Table 9 presents data comparing perceived freedom in leisure and multifaceted leadership orientation scale scores according to individuals' welfare levels. Regarding perceived freedom, no significant difference is observed among individuals of different welfare levels (f = 2.350, *p* = 0.071). Similarly, no significant differentiation was detected in the domains of political leadership and human resources leadership. However, in the charismatic leadership dimension, individuals with poor welfare levels demonstrated a score (M = 15.56) that showed a significant increase compared to the moderate welfare group (M = 15.25) (*p* = 0.004), while individuals with good welfare levels achieved an even higher score (M = 16.26); this interestingly indicates that charismatic leadership levels are higher for the good welfare group when compared to the poor welfare group. A similar situation exists in the structural leadership dimension, where individuals with poor welfare levels scored significantly higher (M=12.68) compared to those at moderate levels (M = 12.30) (*p* = 0.009). Overall, these findings reveal complex and multilayered relationships between individuals' welfare status and their perceived freedom and various leadership orientations.

**Table 9:** Comparison of perceived freedom in leisure and multifaceted leadership orientation scale scores according to individuals' welfare level.

**Subdimensions**	**Welfare level**	***N***	**M ±Sd**	**f**	***p***	**LSD**
Perceived freedom in leisure	Poor^a^	77	102.40 ± 6.26	2.350	0.071	***-***
	Moderate^b^	169	101.36 ± 6.00			
	Good^c^	279	101.43 ± 5.60			
	Very good^d^	108	102.91 ± 5.28			
Political leadership	Poor^a^	77	14.79 ± 3.53	2.569	0.053	***-***
	Moderate^b^	169	14.90 ± 4.81			
	Good^c^	279	15,60 ± 3,34			
	Very good^d^	108	14,51 ± 4,04			
Human resource leadership	Poor^a^	77	15,23 ± 3,46	1,016	0.385	***-***
	Moderate^b^	169	15.30 ± 4.57			
	Good^c^	279	15.88 ± 3.54			
	Very good^d^	108	15.63 ± 4.25			
Charismatic leadership	Poor^a^	77	15.56 ± 3.98	4.482	***0.004***	***b < c*** ***c>d***
	Moderate^b^	169	15.25 ± 4.43			
	Good^c^	279	16.26 ± 4.02			
	Very good^d^	108	14.66 ± 4.36			
Structural leadership	Poor^a^	77	12.68 ± 3.01	3.904	***0.009***	***b < c*** ***b < d***
	Moderate^b^	169	12.30 ± 3.53			
	Good^c^	279	13.26 ± 3.02			
	Very good^d^	108	13.29 ± 2.95			

M, Mean; Sd, Standard Deviation; f, F-value; p, *p*-value; LSD, Least Significant Difference. *p* < 0.05. Bold numbers are used to emphasize significant differences. Letters were used to indicate the differences between the variables on the left side of the tables.

Analysis of Table 10 reveals significant correlations between the perceived freedom in leisure scale and various subdimensions of the multifaceted leadership orientation scale: a low positive significant correlation with political leadership (r = 0.096, *p* = 0.016); a low positive significant correlation with human resources leadership (r = 0.078, *p* = 0.049); a low positive significant correlation with charismatic leadership (r = 0.102, *p* = 0.019); a low positive significant correlation with structural leadership (r = 0.096, *p* = 0.016); and a high positive significant correlation with multifaceted leadership orientations (r = 0.848, *p* < 0.001).

**Table 10:** Correlation analysis between perceived freedom in leisure scale and multifaceted leadership orientation scales.

		**1**	**2**	**3**	**4**	**5**	**6**
Perceived freedom in leisure^a^	r	1					
	p						
	n	633					
Political leadership^b^	r	0.096^*^	1				
	p	0.016					
	n	633	633				
Human resource leadership^c^	r	0.078^*^	0.660^**^	1			
	p	0.049	*** < 0.001***				
	n	633	633	633			
Charismatic leadership^d^	r	0.102^*^	0.666^**^	0.654^**^	1		
	p	0.019	*** < 0.001***	*** < 0.001***			
	n	633	633	633	633		
Structural leadership^e^	r	0.096^*^	0.437^**^	0.537^**^	0.534^**^	1	
	p	0.016	*** < 0.001***	*** < 0.001***	*** < 0.001***		
	n	633	633	633	633	633	
Multifaceted leadership^f^	r	0.848^**^	0.121^**^	0.124^**^	0.117^**^	0.114^**^	1
	p	*** < 0.001***	0.002	0.002	0.003	0.004	
	n	633	633	633	633	633	633

r, Correlation coefficient; p, *p*-value; n, Sample size. ^*^p < 0.05, ^**^p < 0.001. Bold numbers are used to emphasize significant differences. Letters were used to indicate the differences between the variables on the left side of the tables.

Examination of Table 11 indicates that the model constructed between the perceived freedom in leisure scale and the score of multifaceted leadership orientation scale presents a significant relationship (r = 0.849, r^2^ = 0.721; *p* < 0.01). When analyzing the *t*-test results regarding the significance of the regression coefficient, it was observed that the level of perceived freedom in leisure predicts multifaceted leadership orientation (t = 10.530, *p* = 0.000) and explains 72.1% of the variance (f = 318.697, *p* < 0.01).

**Table 11:** Regression analysis of perceived freedom in leisure level predicting multifaceted leadership orientation values.

**Predictor**	**B**	**Std error**	**β**	**t**	***p***
Constant	22.794	2.165		10.530	< 0.001
Multifaceted leadership	1.019	0.026	0.849	39.512	< 0.001

R = 0.849; R^2^ = 0.721.

F = 318.69; *p* < 0.001.

## Discussion

The findings of this research reveal the relationships between perceived freedom levels in leisure time and multifaceted leadership orientations among individuals engaged in outdoor sports. When examined from a gender perspective, the absence of significant differences in perceived freedom levels during leisure time indicates that individuals involved in outdoor sports share similar perceptions of freedom. This finding demonstrates consistency with previous research (Lapa and Agyar, 2012; Serdar and Ay, 2016; Gürbüz and Henderson, 2014; Chen et al., 2013) and supports the gender equality perspective in outdoor sports. While some studies have found higher male participation in leisure activities, this reflects a different dimension related to participation rates rather than perceived freedom levels (Demir and Demir, 2006).

Regarding multifaceted leadership orientations, males achieving higher scores in political leadership aligns with literature findings on men's task-oriented leadership styles (Eagly and Johnson, 1990). However, the narrowing gender gaps in leadership effectiveness represents a noteworthy development (Paustian-Underdahl et al., 2014). The finding that males also scored higher in human-oriented and charismatic leadership challenges traditional stereotypes and suggests the developmental effects of outdoor sports on these characteristics. The absence of gender differences in structural leadership is consistent with Bolman and Deal's (2017) model.

The results obtained regarding marital status reveal that single individuals possess higher perceptions of leisure freedom compared to married individuals. This finding parallels Akyüz and Türkmen's (2016) research and can be explained by single individuals having greater flexibility in time management. The more restricted leisure time perception among married individuals can be evaluated within the framework of Stebbins (2012) serious leisure theory. The constraints imposed by family responsibilities may affect participation in recreational activities such as outdoor sports.

In terms of leadership orientations, married individuals achieving higher scores in political leadership, human resources leadership, and charismatic leadership differs from Gürer's (2012) findings. This divergence may stem from sample group characteristics or study context. The superiority of married individuals in these leadership areas can be explained within (Bolman and Deal, 1991) multifaceted leadership theory framework through the responsibilities and experiences that married life brings. The high performance in human resources leadership, in particular, can be associated with the development of family communication and empathy skills (Yilmaz and Karahan, 2010). Regarding charismatic leadership, this can be explained by the increase in life experiences and development of responsibility consciousness, as suggested by Avolio and Gardner (2005).

When examining the age variable, the absence of significant differences in perceived freedom levels during leisure time is consistent with Lapa and Agyar's (2012) findings. However, the divergence from Serdar and Ay's (2016) finding that freedom perception increases with age may result from sample characteristics. Regarding leadership orientations, the presence of significant differences among age groups in the human resources leadership dimension, with the 29–39 and 40–50 age groups scoring higher than the 18–28 age group, aligns with Avolio et al.'s (2009) findings that leadership skills develop with age. The highest scores in political leadership achieved by the 40–50 age group also support Day et al.'s (2014) findings that political skills develop with age.

Concerning educational status, the absence of significant differences in perceived freedom levels during leisure time is consistent with various studies in the literature (Lapa and Agyar, 2012; Serdar, 2021). This indicates that educational level is not a determining factor in leisure freedom perception. However, high school graduates achieving lower scores in all leadership dimensions emphasizes the critical role of education in leadership skill development. These findings parallel studies by Bass and Avolio (1994), House and Aditya (1997), and Northouse (2018) that emphasize the importance of the educational process in developing leadership competencies.

The findings obtained regarding sports disciplines demonstrate that different outdoor sports affect individuals' freedom perception and leadership characteristics at varying levels. Canoeing achieving the highest score in leisure freedom can be explained by this sport providing individuals with a greater sense of freedom due to its inherent nature (Ewert and Sibthorp, 2014). Orienteering's prominence in charismatic and structural leadership can be associated with this sport's development of individual decision-making abilities and systematic thinking and organizational skills (Priest and Gass, 2018; McKenzie, 2003).

The results obtained regarding participation duration reveal that long-term participation positively affects both leisure freedom perception and leadership orientations. This aligns with Cotterill and Fransen's (2016) findings emphasizing the importance of experience acquisition in leadership development. Consistent with Kleiber et al.'s (1986) findings that leisure activities provide psychological relaxation and personal control, long-term participation in outdoor sports appears to enhance freedom perception.

Regarding welfare level, the absence of significant differences in leisure freedom perception supports Kleiber et al.'s (1986) and Iso-Ahola's (1997) findings that the benefits derived from activities are more important than economic status. Those with good welfare levels achieving higher scores in charismatic leadership is consistent with House and Aditya's (1997) and Northouse's (2018) findings that economic security supports confidence and leadership characteristics. Those with poor welfare levels achieving higher scores in structural leadership can be explained by Pearce and Sims' (2002) and Hackman and Wageman's (2005) findings that difficult economic conditions develop structural leadership skills.

The correlation and regression analysis results reveal a strong and positive relationship between perceived freedom levels in leisure time and multifaceted leadership orientations. This relationship explaining 72.1% of the variance demonstrates the powerful effect of freedom perception on leadership behaviors. Van Dierendonck's (2011) findings that leadership style affects freedom perception and Bass and Avolio's (1994) findings revealing the significant relationship between these two variables support our research results.

In conclusion, it was determined that individuals engaged in outdoor sports possess high levels of perceived freedom in leisure time and moderate levels of leadership orientations. Various demographic and personal variables were observed to affect these perceptions at different levels. The strong positive relationship between perceived freedom levels in leisure time and multifaceted leadership orientations reveals the mutually supportive nature of these two concepts.

This study has certain limitations. Primarily, the sample is restricted to individuals from a specific geographical region and selected on a voluntary basis, which limits the generalizability of the findings. Additionally, since the data were collected through self-report measures, there is a potential risk of social desirability bias in participants' responses.

Future research is recommended to include larger and more diverse samples encompassing different age groups, professional backgrounds, and cultural contexts. Furthermore, the use of qualitative data collection methods (such as in-depth interviews or focus group discussions) may provide a deeper understanding of the relationship between leadership orientation and perceived freedom in leisure time. Longitudinal studies could also be conducted to examine how these variables interact and evolve over time. Lastly, comparative studies that explore the effects of various sports disciplines on individuals' leadership orientations and perceptions of freedom would make valuable contributions to the literature.

- Considering that regular participation in outdoor sports contributes to individuals' leadership behaviors, increasing such activities in university programs could be encouraged.
- In light of the findings indicating that the level of freedom individuals perceive in their leisure time is associated with leadership tendencies, it may be recommended to consider activities involving nature sports as a tool in the field of personal development.
- Given that recreational activities provide not only physical but also psychosocial benefits, nature sports could be structured to support individuals' perceptions of freedom and leadership competencies.
- In future studies, examining the differences between freedom perception and leadership orientations according to different types of nature sports could provide more in-depth contributions to the literature.

## Data Availability

The original contributions presented in the study are included in the article/supplementary material, further inquiries can be directed to the corresponding author.
